# Modelling variations of emergency attendances using data on community mobility, climate and air pollution

**DOI:** 10.1038/s41598-023-47857-4

**Published:** 2023-11-23

**Authors:** Dirk Weismann, Martin Möckel, Heiko Paeth, Anna Slagman

**Affiliations:** 1 Intensive Care Unit, Department of Internal Medicine I, University Hospital of Wuerzburg, University of Wuerzburg, Oberdürrbacherstr. 6, 97080 Würzburg, Germany; 2 Departments of Emergency and Acute Medicine, Campus Mitte and Virchow-Klinikum, Charite—Universitätsmedizin Berlin, Berlin, Germany; 3 Geographical Institute, University of Wuerzburg, Wuerzburg, Germany

**Keywords:** Environmental health, Preventive medicine, Environmental impact, Cardiovascular diseases, Reproductive disorders, Respiratory signs and symptoms, Preclinical research

## Abstract

Air pollution is associated with morbidity and mortality worldwide. We investigated the impact of improved air quality during the economic lockdown during the SARS-Cov2 pandemic on emergency room (ER) admissions in Germany. Weekly aggregated clinical data from 33 hospitals were collected in 2019 and 2020. Hourly concentrations of nitrogen and sulfur dioxide (NO2, SO2), carbon and nitrogen monoxide (CO, NO), ozone (O3) and particulate matter (PM10, PM2.5) measured by ground stations and meteorological data (ERA5) were selected from a 30 km radius around the corresponding ED. Mobility was assessed using aggregated cell phone data. A linear stepwise multiple regression model was used to predict ER admissions. The average weekly emergency numbers vary from 200 to over 1600 cases (total n = 2,216,217). The mean maximum decrease in caseload was 5 standard deviations. With the enforcement of the shutdown in March, the mobility index dropped by almost 40%. Of all air pollutants, NO2 has the strongest correlation with ER visits when averaged across all departments. Using a linear stepwise multiple regression model, 63% of the variation in ER visits is explained by the mobility index, but still 6% of the variation is explained by air quality and climate change.

## Introduction

As a consequence of the economic lockdown^1^ during the SARS-CoV2 pandemic in 2020^2^ a reduction of air pollution and a possible beneficial effect on health was hitherto described in several regions of the world^3–5^. Stroke, ischemic heart disease (IHD)^6^, COPD, acute lower respiratory infections (LRI) and lung cancer are major diseases that are affected by air pollution^7, 8^ and a recent Canadian study also demonstrated an increased number of ER visits for nervous system disorders in association with decreased air quality^9^.

Industrial emissions, traffic related fuel combustion or road dust are the prime sources of ambient air pollution, contributing to nitrogen and sulfur dioxide (NO2, SO2) emissions and to the generation of particulate matter (PM). Clearly, detrimental health effects are related to long-term exposure to air pollution^10–14^. Early reports on air pollution and mortality date back to the 1950s, analysing the harm of the London fog at these days^15^. Over the last decades, a growing body of evidence has proven the impact of air pollution, especially PM concentration, on mortality^16–18^. The attributable burden of disease (BoD) from air pollution is a matter of discussion, but methodological improvements consolidated the rank of air quality as one of the top global risk factors in public health^7^.

While short-term exposure-mortality associations are considerably lower compared to long-term exposures, variations have been observered with a 10 mg/m^3^ increase of PM10 (PM < 10 µm diameter) on a 3-day lag in time-series analysis^19^ and a study from Turkey showed that PM10 and/or SO2 short-term exposure in single- and multi-day lag models was related with increased asthma, and/or COPD hospital admissions^20^.

While both, ambient and indoor emissions, contribute to air pollution^21^, a specific reduction in ambient air pollution was inadvertently achieved by the economic lockdown^22^. Regarding the lockdown in Europe, a large reduction in NO2 concentrations, a less pronounced reduction in PM concentrations and a mitigated effect on ozone concentrations was described^5^. The latter was attributed to non-linear chemical effects in the atmosphere. Overall, however, the total number of deaths attributable to air pollution has not extensively increased in the European region^7^. In Germany, a uniform reduction of ER visits was noted already before, but in particular with the enforcement of the lockdown^23^. This was especially true for patients suffering from acute coronary syndrome, ischemic stroke, and acute exacerbated chronic obstructive pulmonary disease (AECOPD). However, a gradual increase in ER-visits was noted already before the first lockdown ended^23, 24^.

In this study, we aimed to analyse the impact of reduced mobility, changed air quality and weather anomalies on ER-admissions during the first and second lockdowns in the year 2020 compared to 2019. Using 2019 as a reference year is characterized by unusually warm and dry conditions in spring and summer. Yet, the ER-Admission data is only available for 2019 and 2020. Therefore, we had to restrict our analysis to the pre-Corona year 2019 as a reference and 2020 as the year with the most effective lockdowns. We hypothesized, that significant changes in air quality may impact on the incidence of ER-visits for diseases attributable to short-term changes in air quality. The study is based on a cross-validated statistical model combined with a Monte Carlo approach that can identify robust predictors for ER-admissions and their relative importance.

## Results

### Geospatial characteristics

**Figure 1:**
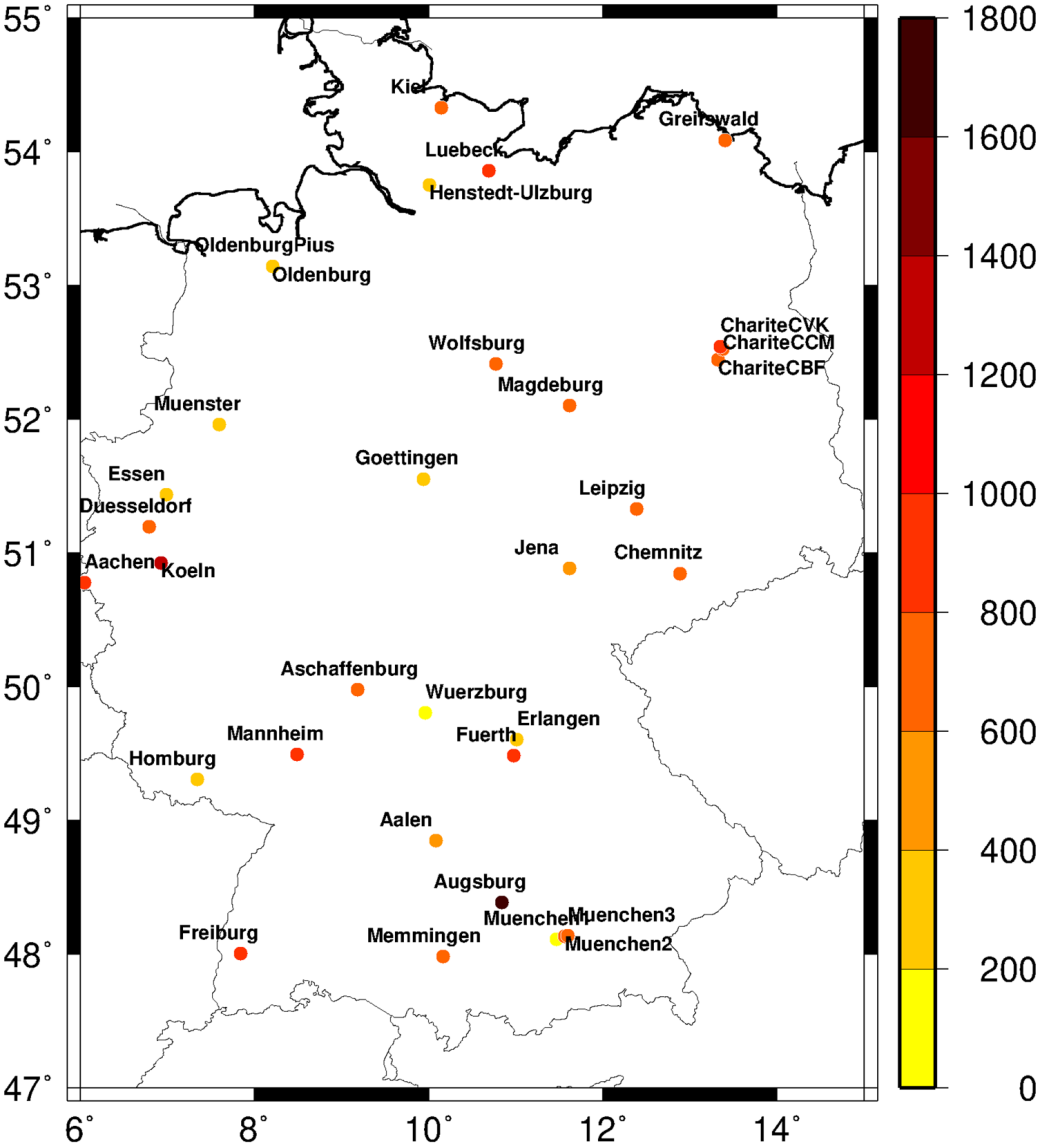
Location of the considered 33 emergency departments across Germany and their mean weekly number of admissions in the years 2019 and 2020. The map was created with the Generic Mapping Tools (GMT Version 4.5.6, https://www.generic-mapping-tools.org).

The 33 participating emergency departments were evenly distributed all over Germany (Fig. 1). They were mainly located within larger cities or in their vicinity. The mean weekly emergency counts varied between under 200 and over 1600 cases. These differences were related to the regional population density and the catchment area of the departments. They accounted for a considerable portion of the total variance of ER admissions. Therefore, all epidemiological time series have been standardized with respect to the pre-Corona year 2019.

### Cases and lockdown

**Figure 2:**
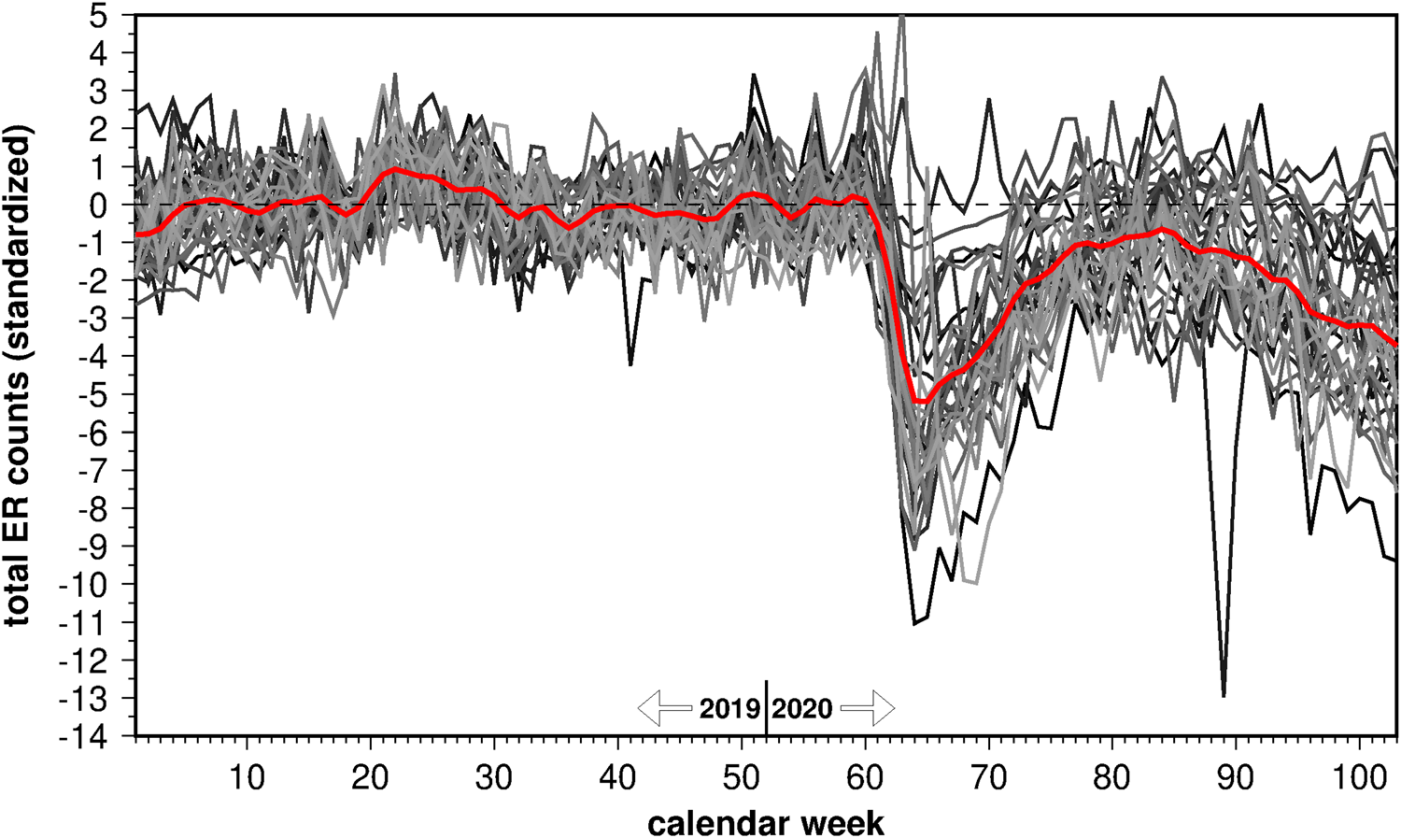
Standardized weekly ER admissions in each individual emergency department (grey lines) and averaged over all 33 departments (red line).

In total, 2,216,627 ER consultations were analysed. A characteristic reduction of case numbers was seen in all but 2 departments, starting in March 2020 (Fig. 2). The mean maximum reduction in case numbers over all departments was enormous and amounts to 5 standard deviations. Details of the ER consultations for this study have recently been published^24^. A gradual increase in summer was observed, followed by a marked decline in autumn. Furthermore, case numbers towards the end of 2020 were lower compared to 2019. Since the first lockdown in March 2020, none of the weeks in 2020 achieved the 2019 level of emergency cases. Except for very few outliers, the time series were rather smooth, especially in 2019. This implies that the low-frequency changes that are presumably imposed by the Corona lockdowns in 2020, clearly stand out from the background noise.

**Figure 3:**
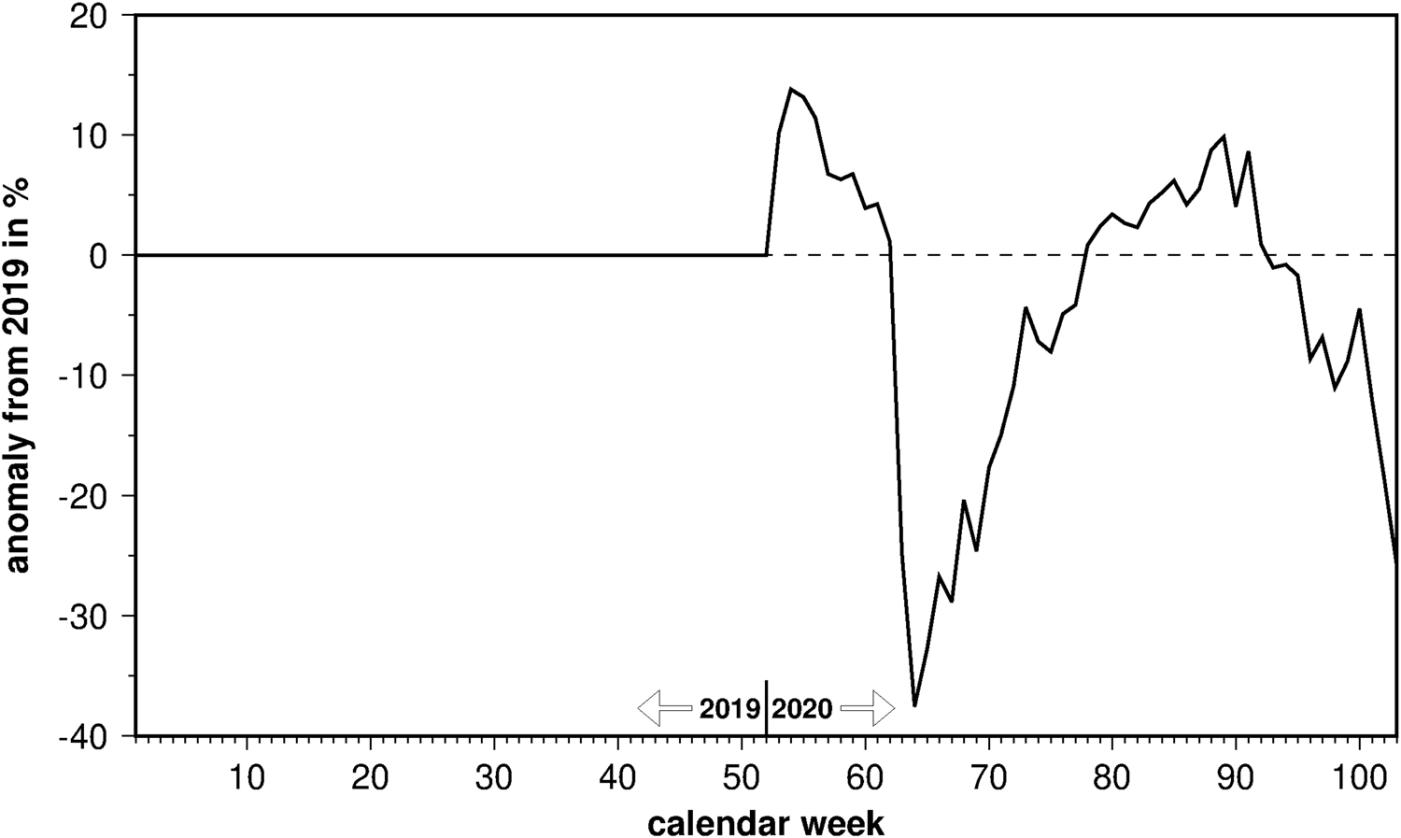
Weekly mobility index of the German population as percentaged difference of a week in 2020 from the respective week in 2019.

The mobility index showed relative weekly anomalies from the year 2019 (Fig. 3). Therefore, no changes occurred in 2019. The year 2020 started with above-normal mobility until the first lockdown was enforced by the German government in March. Within 2 weeks, mobility dropped by almost 40%. After a minimum at the end of March a slow recovery was observed with positive anomalies in summer 2020. With an increasing Sars-Cov2 incidence in autumn and the second lockdown from November onward, the mobility of the German population decreased again, but less rapidly than in springtime.

### Air quality

**Figure 4:**
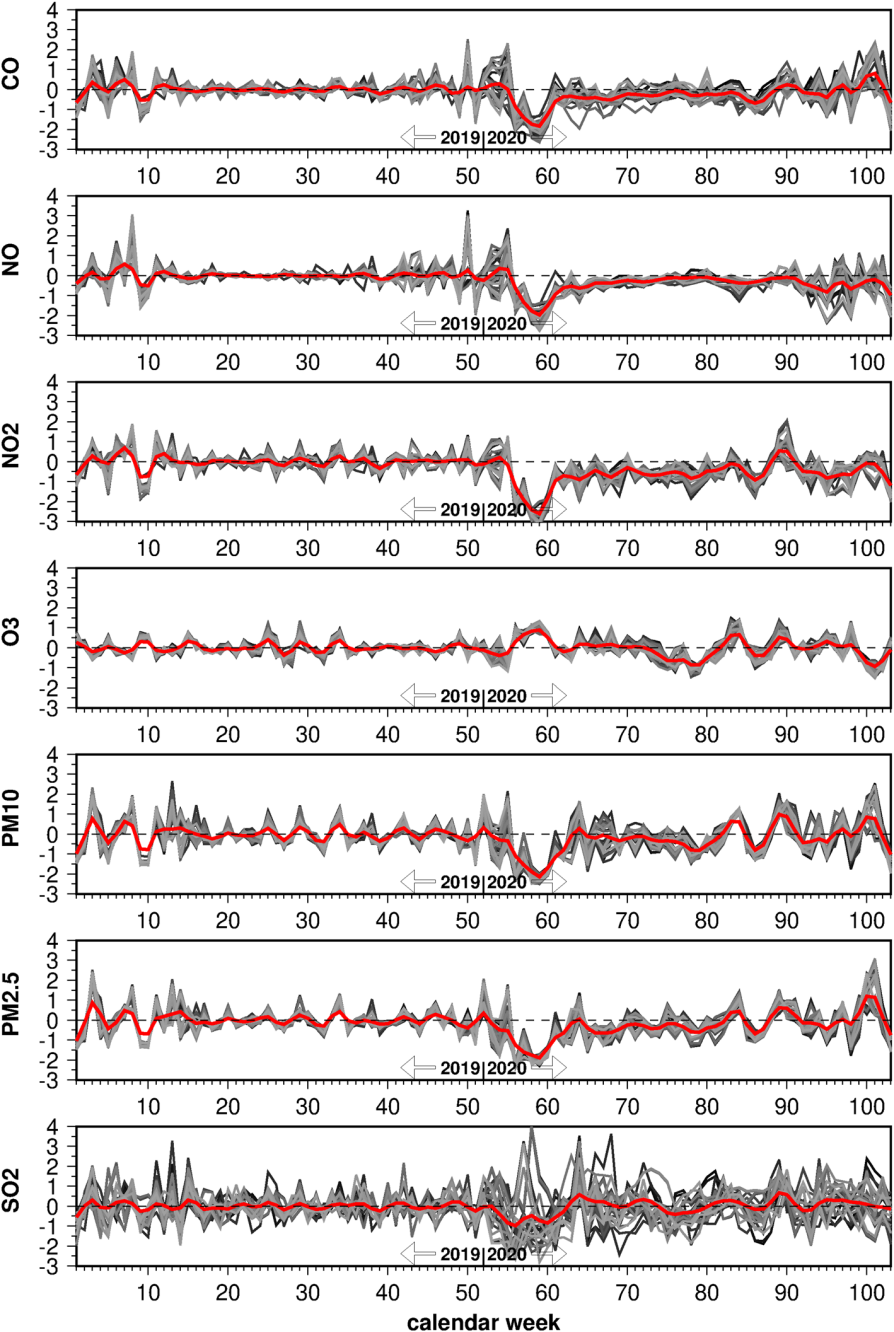
Standardized weekly changes of air pollutants from the 2019 seasonal cycle around each individual emergency department (grey lines) and averaged over all 33 departments (red line).

The standardized time series of the considered air pollutants are illustrated in Fig. 4 as deviations from the seasonal cycle during year 2019. The variability of most pollutants barely differed among the department locations, except for SO_2_, which is more bound to variable local sources. Thus, the mean time series (red lines) represent most of the total variability. They reveal a noticeable minimum burden in February and March 2020, most likely explained by meteorological conditions in the form of intense rainfall and a washing-out effect in the atmosphere. It mainly took place before the first lockdown was enforced. Thereafter, it remained slightly below normal for most of the year. The time series of combustion related pollutants like CO, NO, NO_2_, PM_10_ and PM_2.5_ varied closely in phase with each other. We therefore chose NO_2_ as representative for CO, NO, PM_10_ and PM_2.5_. Note that among all air pollutants NO_2_ had the strongest correlation with emergency cases averaged over all departments (r = 0.3, p = 0.05).

### Weather conditions

**Figure 5:**
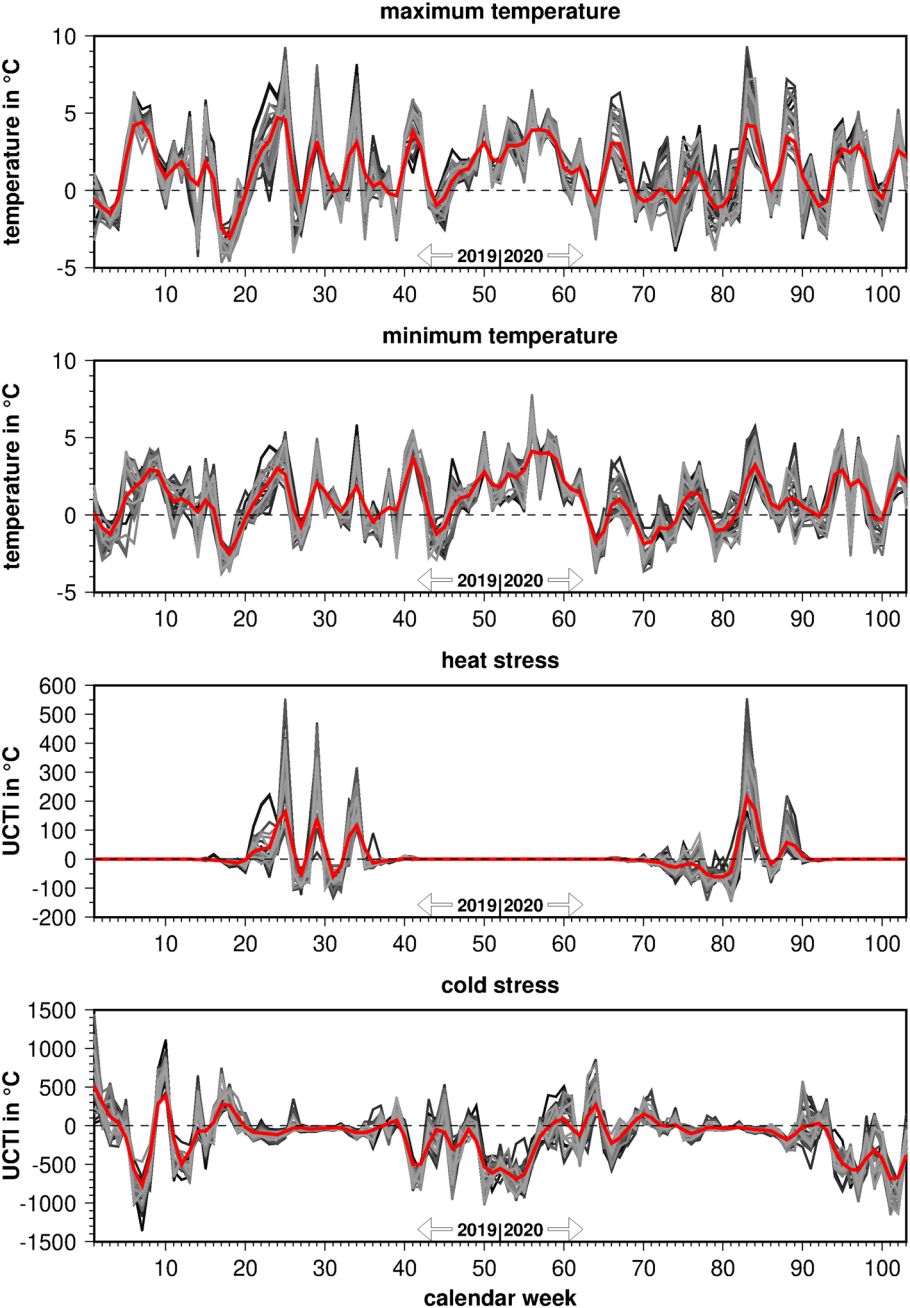
Standardized weekly anomalies of weather conditions from the 1991 to 2020 mean seasonal cycle around each individual emergency department (grey lines) and averaged over all 33 departments (red line).

Figure 5 depicts the standardized time series of daily maximum and minimum temperature as well as cumulative heat and cold stress, aggregated to the same weekly scale as the other predictors and the predictand. The mean seasonal cycle over the 1991–2020 period was removed to highlight anomalously warm and cold weather phases. It is obvious that the winter 2019/2020 has been unusually warm which is visible in the maximum and even more in the minimum temperature (top panels). In addition, cold stress was less pronounced in January and February (bottom panel). Both summers, in 2019 and 2020, were exceptionally hot, with several heat waves lasting up to 4 weeks (second panel from bottom). The temporal variations of weather conditions were well in line across the different regions of Germany because large-scale circulation patterns typically govern the meteorological changes in Central Europe. Note that the existing spatial differences between the weather conditions are eliminated in the course of standardization. Maximum and minimum temperatures are also retained as predictors for the regression model because they exhibit ongoing fluctuations, in contrast to the time series of heat and cold stress, and although they correlate with each other (r = 0.85).

### Prediction of emergency department consultations

**Figure 6:**
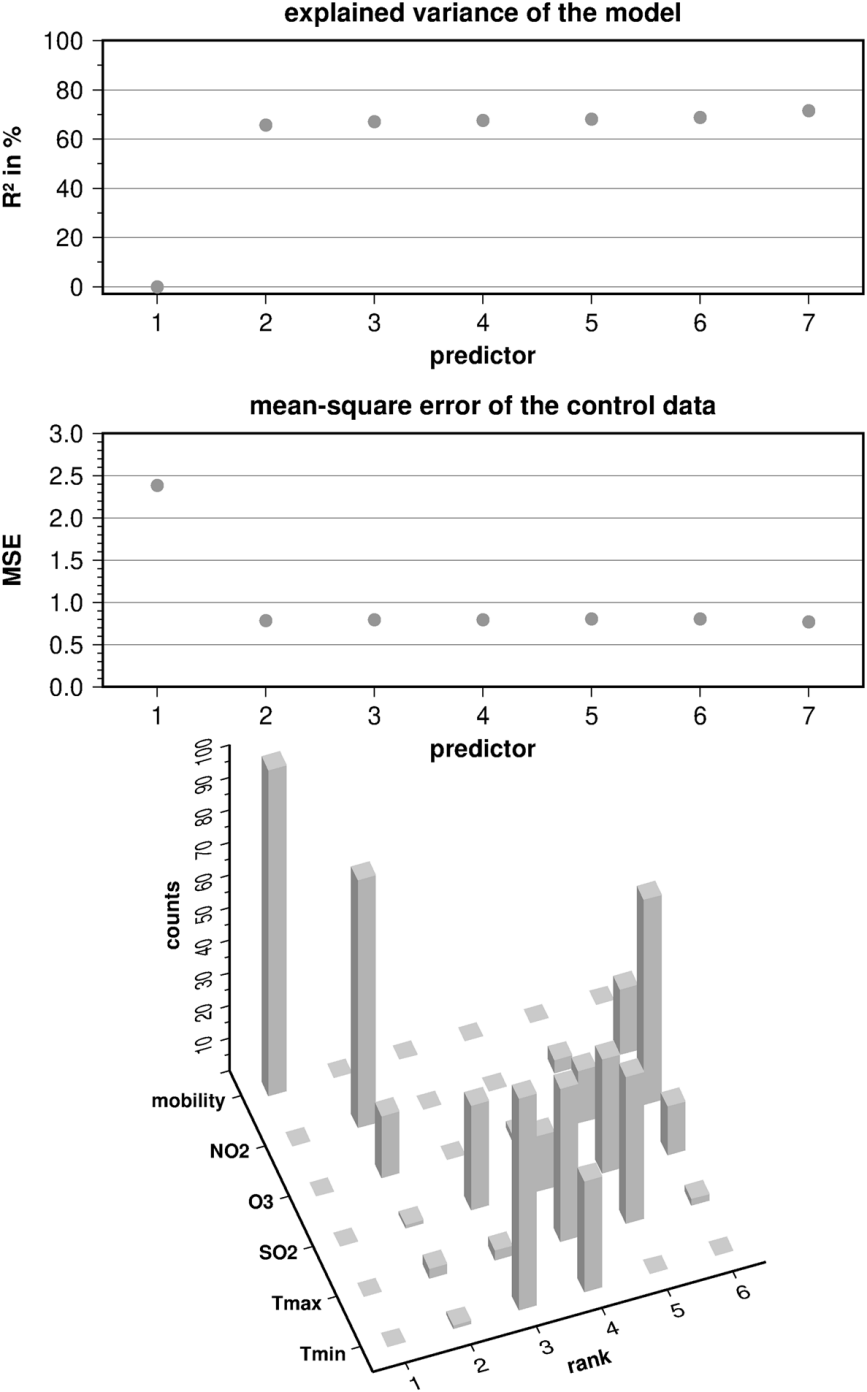
Results of the regression analysis based on an optimal selection of six predictors (see text), averaged over all 33 emergency departments: explained variance by predictor (top), reduction of MSE (mean squared error) by predictor (middle), and rank matrix of the predictors over 100 iterations (bottom).

Together with the uncorrelated mobility index and three representative components of air quality a total of six largely independent predictors were taken into account. Using these six predictors, 69% of the total variability in emergency department consultations could be explained by the multiple regression model based on mean time series over the 33 departments (p < 0.01, Fig. 6, Supplementary Fig. S1). Note that the multiple regression used the long-term mean as default predictor with number 1 in the predictor spectrum. As to be expected, it does not at all contribute to the explained variance of the model (top panel). The prime predictor (here number 2) was the mobility index, which explaining up to 63% of the variability in this model. Air quality and climate changes explained 6% of the variations of ER visits.

The number of robust predictors can be derived from the MSE spectrum in the middle panel of Fig. 6. The MSE dropped substantially when the mobility index was introduced as predictor. With each additional environmental predictor, it decreased only slightly. Nonetheless, the minimum MSE was achieved when all predictors were taken into account, implying that they are all robust with respect to the control data.

The bottom panel of Fig. 6 illustrates the ranking of the predictors over 100 iterations of the statistical model. There is no doubt about the dominant role of the mobility index as predictor for ER visits in the year 2020: it is drawn as leading predictor in each of the 100 iterations. The second rank is mainly assigned to the NO_2_ concentration as an indicator of air quality. In some model iterations, it was the maximum temperature or the tropospheric O_3_ concentration. Minimum temperature covered the third rank, but it was sometimes replaced by the atmospheric SO_2_ burden. The lower ranks are more shared among the predictors. In order to cross-validate our findings, we also evaluated the effect of squaring single terms (e.g. Mobility index) or meaningful combinations (e.g. all air quality predictors; Supplementary Figs. S2–S6). In this analysis, the explained variance ranged between 63.5 and 68.5% (Supplementary Table S1). Additionally, we analysed the effect of PM2.5 instead of SO2 in model 7 (Supplementary Fig. S7). With PM2.5, the explained variance increased to 70.9%, but PM2.5 dropped down to rank 5.

**Figure 7:**
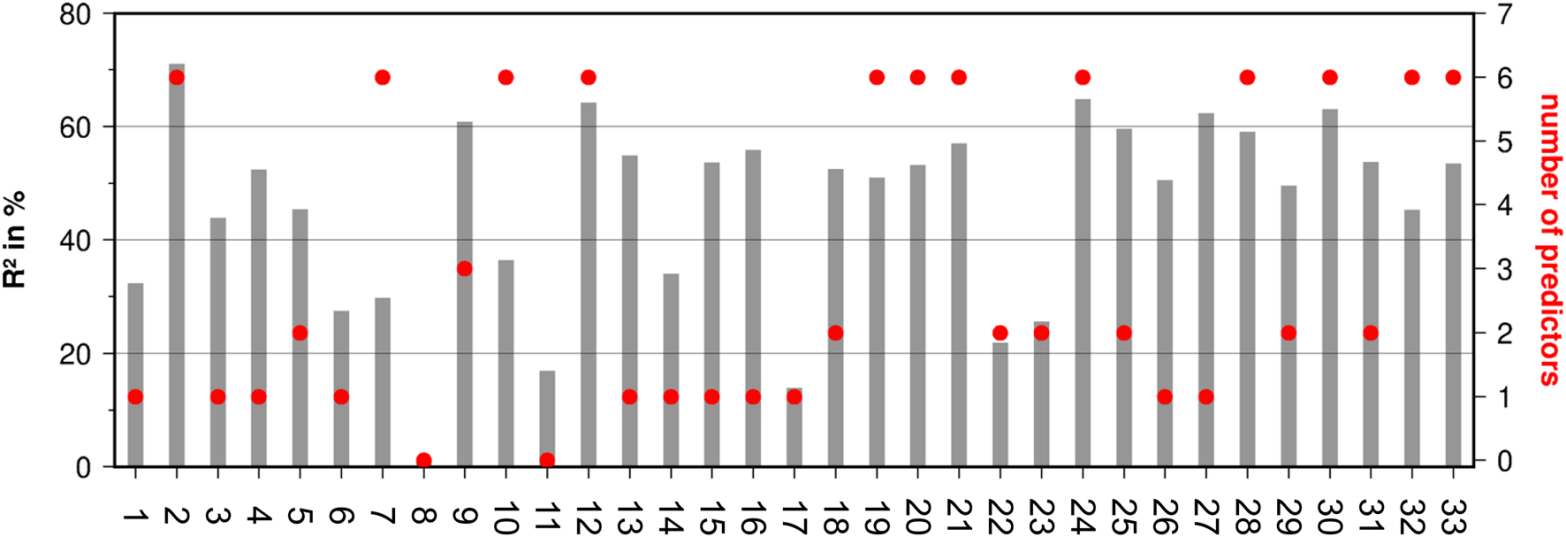
Results of the regression analysis for each of the 33 emergency departments: explained variance of the statistical model (grey bars) and maximum number of robust predictors selected by cross validation out of a total of six predictors (red dots).

When the regression model was applied to individual time series for the 33 departments, the explained variance was slightly reduced due to the higher noise background at single locations (Fig. 7), but it still ranged between 20 and 70% (p = 0.01). An exception was found by the department No 8 (Fig. 7), that barely exhibited any fluctuations over the 2019–2020 period, also missing out the drop after the first lockdown (Fig. 2). Figure 7 also shows that the explained variance is a function of the number of predictors retained by cross validation. At most locations, the full spectrum of six predictors was found to be robust. Sometimes the model only relied on the mobility index, but still achieved a good performance (Center No 27, Fig. 7), sometimes even the mobility index was not a good predictor (center No 17), and in one case only the long-term mean was identified as a robust predictor which, however, accounted for 17% of total variance (No 11, Fig. 7). In summary, the suggested statistical model with six representative predictors of emergency cases is well corroborated on the basis of individual locations and on average over all considered departments across Germany.

## Discussion

The social an economic lockdown was associated with a significant decrease in emergency attendance in Germany^24^. To better understand the impact of lockdown measures on variations of emergency attendance, we analysed the contribution of community mobility, air quality and weather changes. We can show that population mobility predicts 63% of the variation in emergency visits in Germany, while lower air pollution concentration explains another 6%.

Improved air quality in turn has been shown to decrease mortality and has been linked to cumulative exposure of PM2.5 e.g. in Eastern Germany^25^. Although air quality has improved considerably during 5 years in Bejing, China, daily changes in PM2.5 concentration are still associated with hospital admissions^26^. Analysis for Europe suggest a short-term increase in mortality by 0.76% for each increase in PM10 by 10 µg/m^3^^27^.

Great impact of suddenly improved air quality as a result of economic closure in 2020 have been reported from parts of the world^28, 29^. For example, relative reductions in PM 2.5 were greater in Europe, but the actual reduction in PM 2.5 concentrations was more pronounced in China^4^. In addition, PM 2.5 concentrations in Europe are reported to be three times lower than in China^4^. In particular, the 2 years included in this analysis, 2019 and 2020, were classified as exceptionally clean in Germany, with the lowest number of days on which the limit values for NO2, SO2 and PM10 were exceeded^30^. In contrast, ozone is not dependent from emissions but rather from chemical reactions of organics and NO2 in association with intensive sun light exposure^31^. Therefore, rising ozone levels reflect longer episodes of increased air pollution in combination with an anticyclone. The WHO target of ozone concentrations below 100 µg/m^3^ as an 8-h moving average was missed at almost all ground stations in Germany in 2020. The need for an integrated view using models that account for multiple pollutants to determine the health effects of air pollution is controversial^13^. The interactions among pollutants reflected in the formation of ozone are difficult to capture. Furthermore, reports suggest that in addition to size and concentration, the composition of particulate matter is also important^32^. In line with other studies^3^, we observed a high correlation of pollutants and NO2 was selected as representative for CO, NO, PM 10 and PM 2.5. High NO2 concentrations were associated with mortality^3^, with an 10 µg/m^3^ increase in NO2 concentration resulting in a RR of 1.03 [1.01–1.04] for COPD mortality^13^. In addition, NO2 has been speculated to contribute to SARS-CoV2 morbidity, whereas particulate matter chronically impairs pulmonary integrity^28^. Elevated SO2 concentrations during the lockdown have also been reported^3^, indicating that the improvement in air quality was not uniform worldwide. These authors also showed, that SO2 concentrations varied across the country. By modeling the number of ER visits and changes in air quality, we were able to account for such regional differences. Compared to PM2.5, SO2 had a greater impact in our analysis achieving higher ranks after iteration of the models (Fig. 6, Supplementary Fig. S7). Therefore, SO2 was chosen as predictor over PM2.5, since PM2.5 was also highly correlated with NO2 and NO2 was chosen as representative for CO, NO, PM_10_ and PM_2.5_. As the explored links between lockdown, air quality, weather conditions and emergency cases in Germany are unknown, we have preferred to employ the least complex statistical model using linear relationship to avoid overfitting of the model. A nonlinear regression model may enhance the explained variance, yet it is not well founded in theory. Taken together, a 6% decrease in emergency visits is an important observation on this basis and the achieved explained variance of the statistical model is relatively high compared with other studies in the nexus between environmental and medical issues^33, 34^.

Short-term effects of air pollution have been examined, and variations within 1 to 3 days have been shown to have effects on morbidity and mortality^19^, as have medium-term effects extending over weeks^19, 35^. A study from Denmark suggests, that daily survival in myocardial infarction was associated with traffic-related air pollution^36^. Also, a significant increase in blood pressure was associated with an increase in local PM2.5 concentrations in a study with 347 adults^37^. In addition, seasonal variations in air pollution have been associated with mortality in hospitalized patients with cardio-respiratory diseases^38^. While this is in support of our study, in contrast, there was a large short-term decrease in NO2 and PM10 concentrations during weeks 10 and 11 in 2019 as a result of storms Bennet, Cornelius, Dragi, Eberhard, Franz, Heinz and Igor passing over Europe. We analysed the effect of these storm-related air quality improvements in detail but did not detect any changes in hospital admissions during weeks 10 and 11 and the two weeks thereafter. Possibly, the weekly aggregation of the admission data in our study does not provide the resolution required to identify variations in this regard. Recent data suggest that air-pollution analysis needs to be performed at neighbourhood-level resolution^39^. With respect to an exposure–response relationship, we correlated air pollution data from a 30-km^2^ grid around each emergency department to account for regional differences. However, population density was not included in the analysis but is indirectly reflected in the average admissions per emergency department. Since also other studies suggest a considerable seasonal and regional variation in health effects, it was hypothesized, that community-specific relative rates could be explained by differences in the chemical composition of particulate matters^32^.

Ambient air pollution was associated with emergency department visits also before the SARS-CoV2-pandemia^40, 41^. Importantly, the number of ER-visits increased steadily during each lockdown, which was associated with a steady increase in community mobility. The widespread use of medical masks and KN95/FFP2 masks in Germany also contributed to reduced exposure to airborne pollutants, but the effect is difficult to assess because masks were primarily worn indoors. Recent studies on masks have focused on the prevention of transmittable diseases, but not on reducing exposition to pollutants. Traffic noise is known to affect health^42, 43^ and indeed the sudden improvement in noise may have contributed to our observation. Obviously, the number of traumas is associated with traffic intensity, but lower traffic volume does not explain the decrease in nontraumatic emergencies. Covid-anxiety clearly plays a role, whereas the overall effects remain unclear^44^. Indeed, the decline in the number of unequivocal emergencies was not as pronounced as that in the number of unclear cases, and conversely, fluctuations in apparent emergencies during the lockdown were only moderate^24^. Reduced availability of general practitioner capacity may also have reduced the number of admissions. It is well described how limited mobility reduces infectious disease transmission^45^, but again, the majority of ER-visits were not related to communicable diseases because we focused on trigger diagnoses such as myocardial infarction, stroke and AECOPD. Reduced mobility may indicate reduced social distress, and one could speculate whether reduced workload has a positive effect on health. However, social distancing itself increases the prevalence of depression and anxiety^44^ and is therefore associated with an increased burden of disease.

Another key determinant of morbidity and mortality are climatic stressors during heat waves and cold spells. The 2003 heat wave in Western and Central Europe has probably caused up to 70,000 deaths in France and Germany^46^. Since then, heat waves in Germany have claimed lives almost every year, especially in the southern part^47^. The occurrence of strokes is particularly sensitive to certain weather anomalies and extremes, e.g., heat waves, cold spells, and rapid changes between air masses of opposite temperature^48^. In addition, weather anomalies interact with air quality^49^ and pose a double risk for various disease patterns such as cardio-vascular disease in European cities^50^.

A correlation between air quality and SARS mortality rates was already identified during the response to the first SARS outbreak in 2003^51^. In addition, an 18% decrease in chronic obstructive pulmonary disease mortality rates in China in 2020 was associated with a reduction in PM 2.5 emissions after the introduction of the lockdown^52^. A stringent lockdown was also enforced in India from March 24, 2020. A significant improvement in the air quality index, which includes particulate matter (PM10 and PM2.5), nitrogen dioxide (NO2), sulfur oxide (SO2), carbon monoxide (CO), and ammonia (NH3), was described from Sentinel-5 satellite data on the first day of the lockdown, and a decrease in mortality was later reported specifically in areas with improved air quality^29^. However, differences in the effect of a lockdown are found in a study comparing changes in air quality in London and Delhi where it turned out, that also other causes such as residential and background pollution are important^53^. The climate in 2019 and 2020 was also not representative for the central European climate, as both years were exceptionally hot in summer. In 2020, the second warmest winter and the second warmest year on record were reported^30^. Lower heating demand is expected to improve air quality, whereas higher ambient temperature could be associated with enhanced chemical reactions in the stratosphere or have a direct negative impact on health. Thus, the overall effect of opposing trends in air quality and climate is unclear, but it appears that the negative effects of rising temperatures are more important^54^. Indeed, heat stress proved to be one of the most important variables in predicting emergency admissions in our model.

A limitation of our study is the short observation period, since climate and air quality studies usually include decades. Assuming, that exposure increases the risk of developing individual diseases, such as lung cancer, long-term studies are needed. However, exposure to pollutants could also accelerate the manifestation of a disease such as AECOPD or lower respiratory tract infection, or promote plaque instability in myocardial infarction. In such models, short-term changes should visibly alter the incidence of related diseases accordingly. In addition, we used long-term climate data to identify significant changes from baseline during the observation period. The short- and long-term effects of changes in air quality are not well understood, but data from other parts of the world show that mortality decreases with increasing air quality^38^. This study focused on adults, but the impact for Children’s Health was recently highlighted in a comprehensive review^55^. Finally, predictand and predictors are appropriately processed to remove confounding effects such as seasonal cycles and shifts between emergency departments.

## Conclusion

In summary, we were able to explain 69% of the variation in ER visits by using community mobility, climate, and air pollution data as independent variables. Community mobility had the greatest impact in this model. While overall air quality in Germany is very good/excellent, still 6% of the variation in emergency attendances could be explained by improvement of air quality during phases of economic lockdown in 2020 in Germany.

## Materials and methods

### Study centers

In this German multicenter study, data were collected in 33 emergency departments participating in the German Forum of University Emergency Departments (Forum universitärer Notaufnahmen, FUN; n = 24) and the German Action Coalition for Information and Communication Technology in Intensive Care and Emergency Medicine (Aktionsbündnis Informations- und Kommunikations-technologie in Intensiv- und Notfallmedizin, AKTIN) Emergency Department Registry (AKTIN; n = 9).

### Data collection

Data collection was conducted as previously described^24^. Data collection included site characteristics of each emergency department as well as specific regional aspects. These included the number of patients and beds in each ED, regional lockdown measures and dates, and information on SARS-CoV2 testing^24^. Anonymized data were aggregated before transmission to the central data management at the Charité—University Hospital Berlin, Germany. The frequency of chronic obstructive pulmonary disease (COPD; ICD-10: J44) was recorded. Diagnoses were assigned to calendar weeks according to date of admission, and the coding of the ICD codes given was considered as the diagnosis, irrespective of whether it was an emergency department diagnosis, main hospital diagnosis, or a secondary diagnosis. The period of data collection covered all calendar weeks in 2019 and 2020. For temporal granularity, weekly intervals were chosen (calendar weeks). No case data were merged on an individual-case basis.

### Method of data extraction and transmission

Data for the 26 participating FUN centres were extracted from the hospital information systems, transferred to Excel spreadsheets in an aggregated manner (case numbers per CW), checked for internal plausibility and then transmitted to the central data management at the Charité—University Hospital Berlin, Germany, in anonymized form. In 10 hospitals, data retrieval of case-related data was carried out centrally via the infrastructure of the AKTIN emergency department registry^56^, which enables multicentre use of routine data irrespective of the local emergency department documentation system^57^.

### Air quality and climate data

Air quality data were provided by the Umweltbundesamt, 06844 Dessau, Germany (UBA 2021). In total, seven components of air pollution were considered: CO, NO, NO_2_, O_3_, PM_10_, PM_2.5_ and SO_2_. The pollutant concentration was measured hourly over the years 2019 and 2020 at over 600 ground level stations throughout Germany in μg/m^3^. In this study, we only used stations with a maximum distance of 30 km around the corresponding emergency department. In this study, we only used stations with a maximum distance of 30 km around the corresponding emergency department. The 30-km criterion is an estimator to determine a representative air quality for the exposed population of the catchment area on the one hand and on the other hand 30 km semi-rural/semi-urban corresponds approximately to a distance that can be reached within 45 min. This should correspond to the core catchment areas of university emergency departments in non-metropolitan areas, which is typical for Germany. The number of available stations is slightly different for each pollutant. The minimum, mean and maximum number of groundstations within a 30 km catchment area around the emergency departments is 1(in only one case), 28.6 and 76, respectively. All pollutant time series have been aggregated to weekly means, in accordance with the clinical data.

Meteorological data were taken from the newest ERA5 reanalysis data set in globally 30 km resolution provided by the European Centre for Medium-Range Weather Forecasts in Reading, UK^58^. It emanates from a numerical weather forecast model that was initialized by all globally available meteorological observations from ground stations, radiosondes and satellites, providing a high-quality, complete and multi-variate dataset for meteorological and climatological assessments^58^. We considered daily maximum and minimum temperature at 2 m height, aggregated to weekly means over the years 2019 and 2020. When comparing the meteorological time series with each other, it turns out that positive anomalies of maximum temperature in summer stands for heat stress and negative anomalies of minimum temperature during the cold seasons for cold stress. In addition, positive anomalies of minimum temperature during summer denote heat stress, e.g., in the form of tropical nights. All correlations were statistically significant at an error level of 1%. In addition, a biometeorological index was considered from the same data set, i.e., the Universal Thermal Climate Index (UTCI). The UTCI is a commonly used index of physiologically relevant cold and heat stress (e.g.^59^). It is based on temperature, air moisture, solar radiation and wind speed. Here, we extracted two variables from the UTCI, indicating the cumulative heat stress and cold stress, respectively, over a calendar week. In order to enhance the spatial representativeness of local meteorological conditions, nine grid boxes around each emergency department have been averaged.

### Mobility data during the lockdowns

The primary effect of the lockdowns during the year 2020 was assessed by means of a mobility index of the German population which represents a metric index that is appropriate for classical statistical models^60^. This index is based on anonymized mobile phone data at a daily time step, aggregated to the national level of Germany. It measures the total displacements of citizens across Germany. It has been introduced by the Federal Statistical Office specifically for the Corona pandemic to assess the effects of the lockdown imposition by the German government.

### Data analysis

The ER admissions varied substantially among the 33 emergency departments, as does the population density and, hence, the number of people living in the catchment area of each emergency department. As these effects were not relevant for the phase relationships between our variables, we have standardized all time-series with respect to the mean and standard deviation of pre-Corona year 2019. The changes were then measured in standard deviations and denote the temporal changes that are of interest in the present study. The mobility index was used as percentage of 2020 with respect to 2019 based on weekly time series. Consequently, this index is constant over 2019 and measured the deviations during the year 2020.

The time series of air quality and regional weather conditions were standardized with respect to 2019 to remove spatial offsets, e.g., between urban and non-urban regions. In addition, they exhibit marked seasonal cycles that dominate the temporal variations and, hence, distract from the lockdown-induced differences between 2019 and 2020 that are of interest here. Most components of air pollution peak in winter when anthropogenic emissions were enhanced, except for tropospheric ozone (O_3_) that had a maximum in summer presumably due to more abundant photochemical reactions in the lower atmosphere. As the air quality data are not available over a longer time window, the seasonal cycle has been estimated by means of a 9-week running mean over the year 2019 that, thereafter, has been subtracted from the entire time series. Thus, the time series of air quality also denote deviations from a typical seasonal cycle in a pre-Corona year. In order to design the model as efficient as possible, only one representative was included in the regression analysis when predictor time series highly correlated with each other. Concerning the climate indices, the seasonal cycle of daily minimum and maximum temperature was even more pronounced. Heat stress only occurs between mid-May and end of August, where cold stress did not happen at all. Due to data availability, a proper mean seasonal cycle at weekly scale has been computed over the 1991–2020 period and subtracted from the 2019–2020 time series which, then, also reflected weekly anomalies of unusually warm or cold weather.

The statistical relationship between the three categories of predictors, i.e. mobility, air quality and weather, on the one hand and ER visits as predictand on the other hand was estimated by means of a linear stepwise multiple regression model^61^. With seven components of air quality, four climate indices and the mobility index, the number of predictors is quite large. To avoid overfitting, the statistical model was based on only six out of 12 available predictors, two for climate, three for air quality and finally the mobility index. The subset has been selected according to the matrix of multicollinearity of the original predictors (Supplementary Fig. S1). The remaining predictors represented the main processes in the assumed link between lockdown, air quality, weather anomalies and emergency cases. In addition, the statistical model itself was able to identify and exclude predictor multicollinearity and, hence, overfitting. For this purpose, all weekly time series were split up into a training data set to fit the regression model and a control data set for cross validation, using the mean squared error (MSE) between the original predictand and the estimated predictand from the model. The whole sample consists of 103 complete calendar weeks in the years 2019 and 2020, 20 of which were retained for the cross validation. The selection of these 20 weeks was managed by a random process and iterated 100 times. Thus, the statistical model was estimated 100 times on the basis of different random samples, according to a classical Monte Carlo approach^61^. In each iteration, the stepwise selection of predictors stopped, when the MSE increased in the control data, indicating that the new predictor was not robust with respect to independent data, mostly because it exhibited multicollinearity with a predictor that has been selected before. This procedure provided the following outcome: a ranking of the predictors over the 100 iterations, the optimal number of robust predictors, the mean explained variance of the statistical model and its statistical significance.

We applied a linear statistical model, since the nexus between lockdown, air quality, climate and ER-Admissions is not backed up by any theory nor by empirical evidence that would justify higher-order relationships. Therefore, we chose a conservative proceeding using a simple model. In addition, nonlinear models require substantially larger samples that are not given by the available ER-Admission data.

### Ethics and data protection

This study was performed in line with the principles of the Declaration of Helsinki. Approval was granted by the Ethics Committee of the Charité—University Hospital Berlin, Germany (EA1/163/20) and the data use and access committee of AKTIN (Project-ID 2021-001). The project represents a merging of aggregated data, which, due to the low temporal granularity, were no longer person-related and, thus, anonymous. The need of informed consent was waived off by the Ethic Committee at the Charité (EA1/163/20).

### Supplementary Information

Supplementary Information.

## Data Availability

The clinical datasets generated during and/or analysed during the current study are not publicly available due to data safety regulations but are available from the corresponding author on reasonable request.
