# Supplementary material for: Modelling variations of emergency attendances using data on community mobility, climate and air pollution
Source: Sci Rep. 2023 Nov 23;13:20595. doi: 10.1038/s41598-023-47857-4 (PMC10667222; doi:10.1038/s41598-023-47857-4)
Supplement: Supplementary file 1 — Supplementary Information. [file 41598_2023_47857_MOESM1_ESM.docx]

Supplemental material


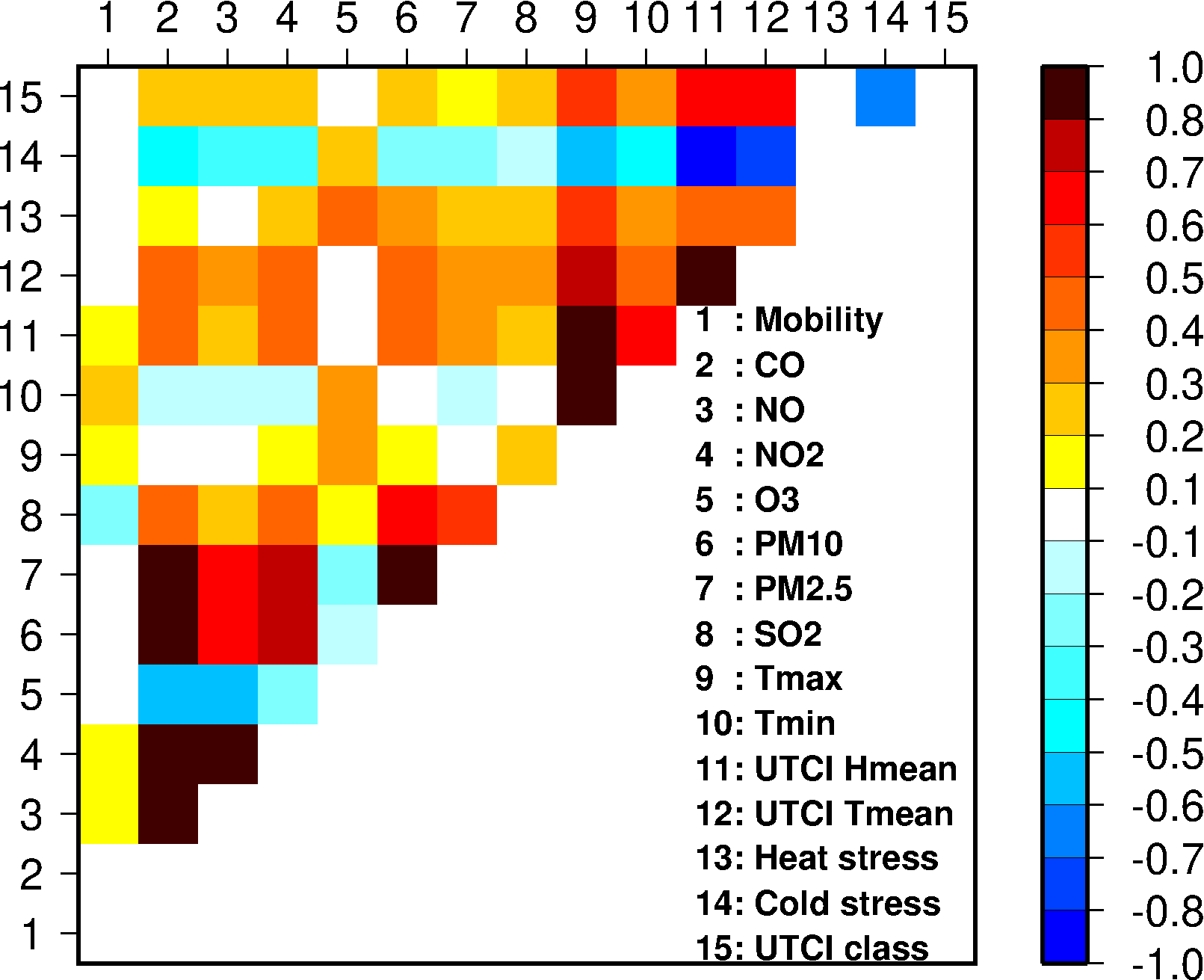


Supplementary Figure S1: A Correlation matrix including all predictors.

Supplementary Table S1: Summary of models with squared predictors

| Model | Explained Variance | Variance explained by air pollution |
| --- | --- | --- |
| Model 1  All predictors were kept linearly | 69.0% | 6% |
| Model 2  Squared: All predictors | 63.6% | 8.6% |
| Model 3  Squared: only Mobility index | 63.5% | 11.3% |
| Model 4  Squared: Air quality predictors | 67.3% | 3.1% |
| Model 5  Squared: climate predictors | 68.5% | 5.4% |
| Model 6  Squared: Climate and air quality predictors | 67.3% | 3.6% |
| Model 7  All predictors were kept linear; SO3 was substituted by PM2.5 | 70.9% | 5.1% |


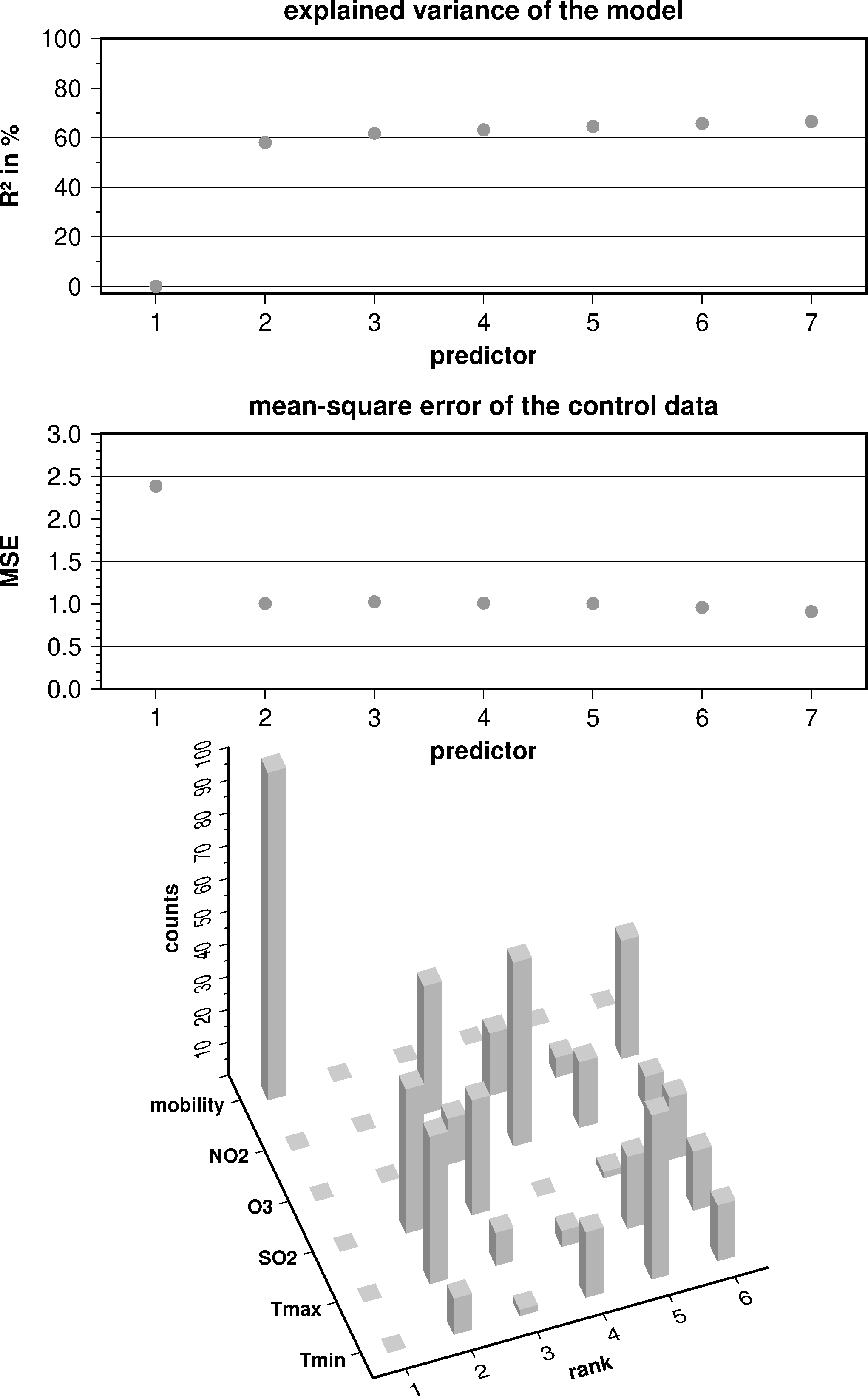


Supplementary Figure S2: In model 2 all predictors were squared.


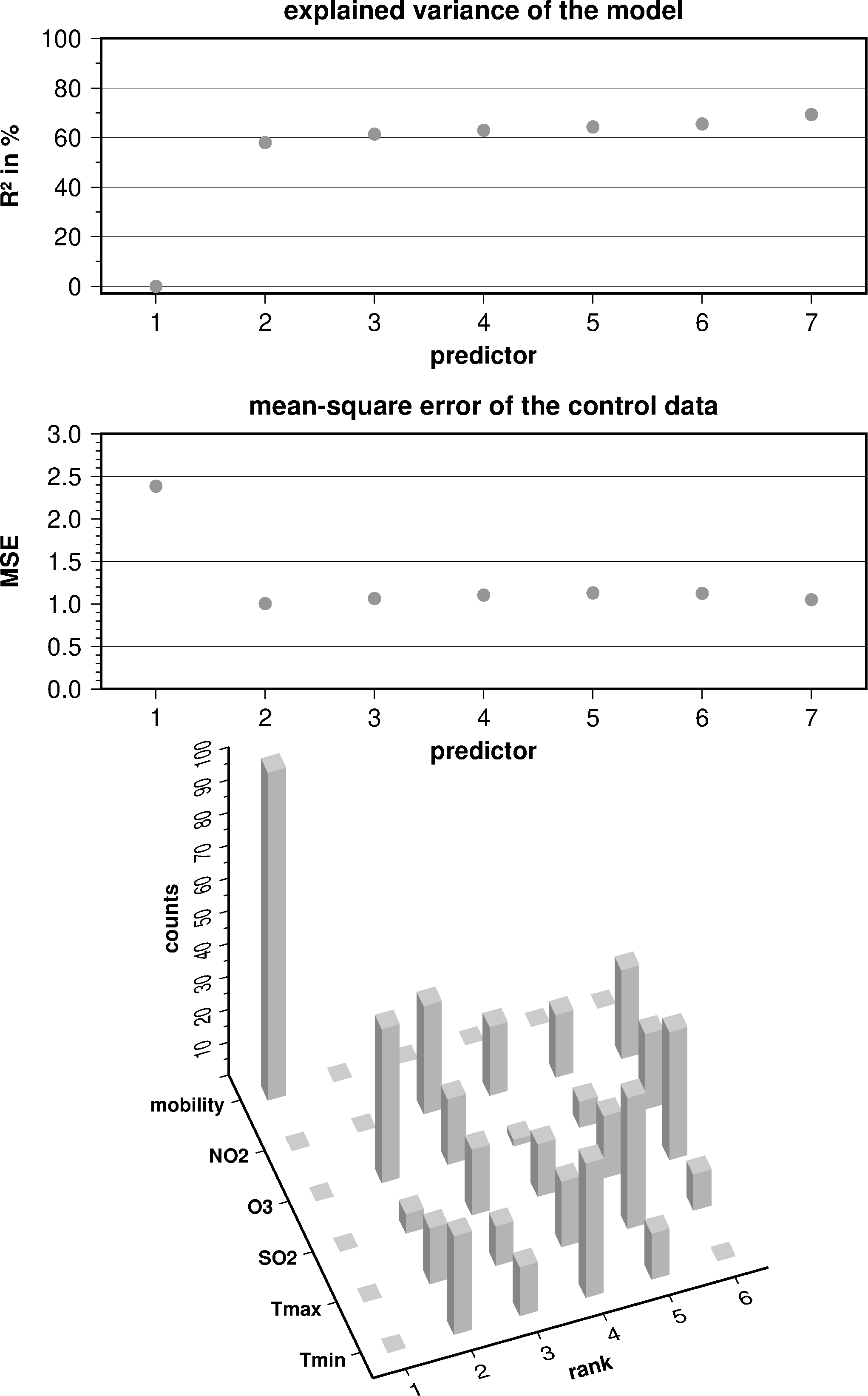


Supplementary Figure S3: In model 3 the mobility index was squared, and all other predictors were kept linearly.


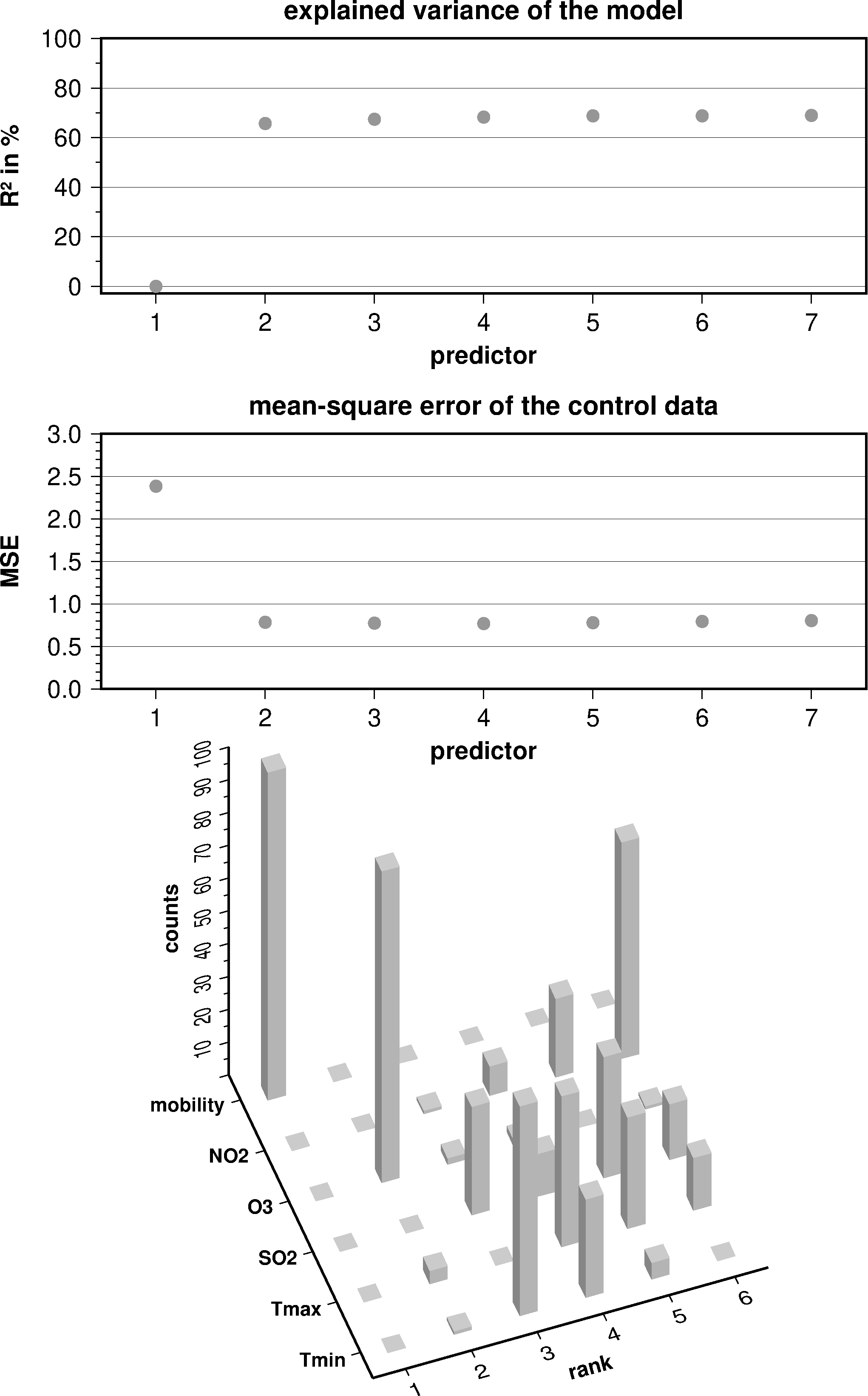


Supplementary Figure S4: In model 4 only predictors for Air quality were squared, and others were kept linearly.


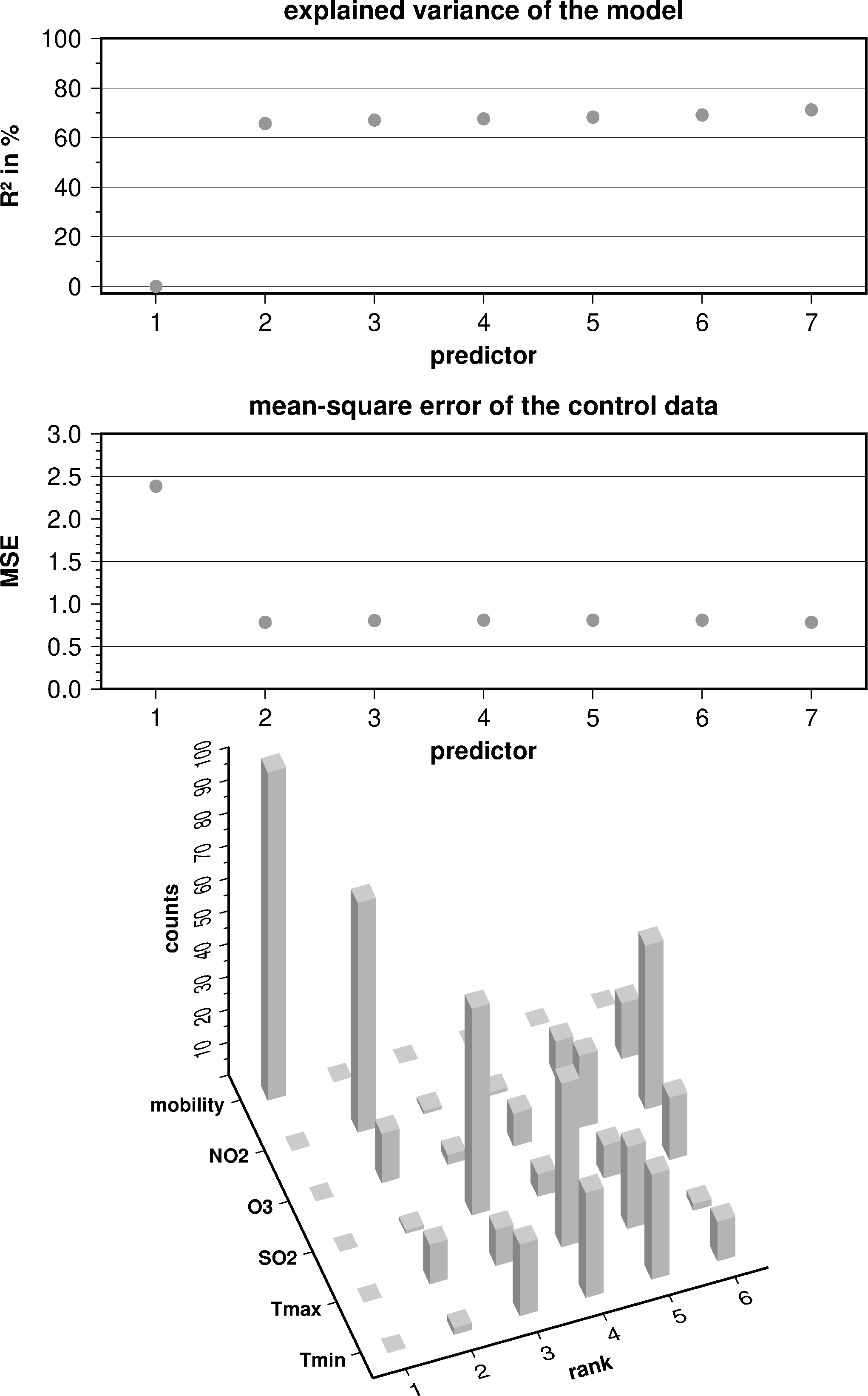


Supplementary Figure S5: In model 5 only climate predictors were squared and all other predictors were kept linearly.


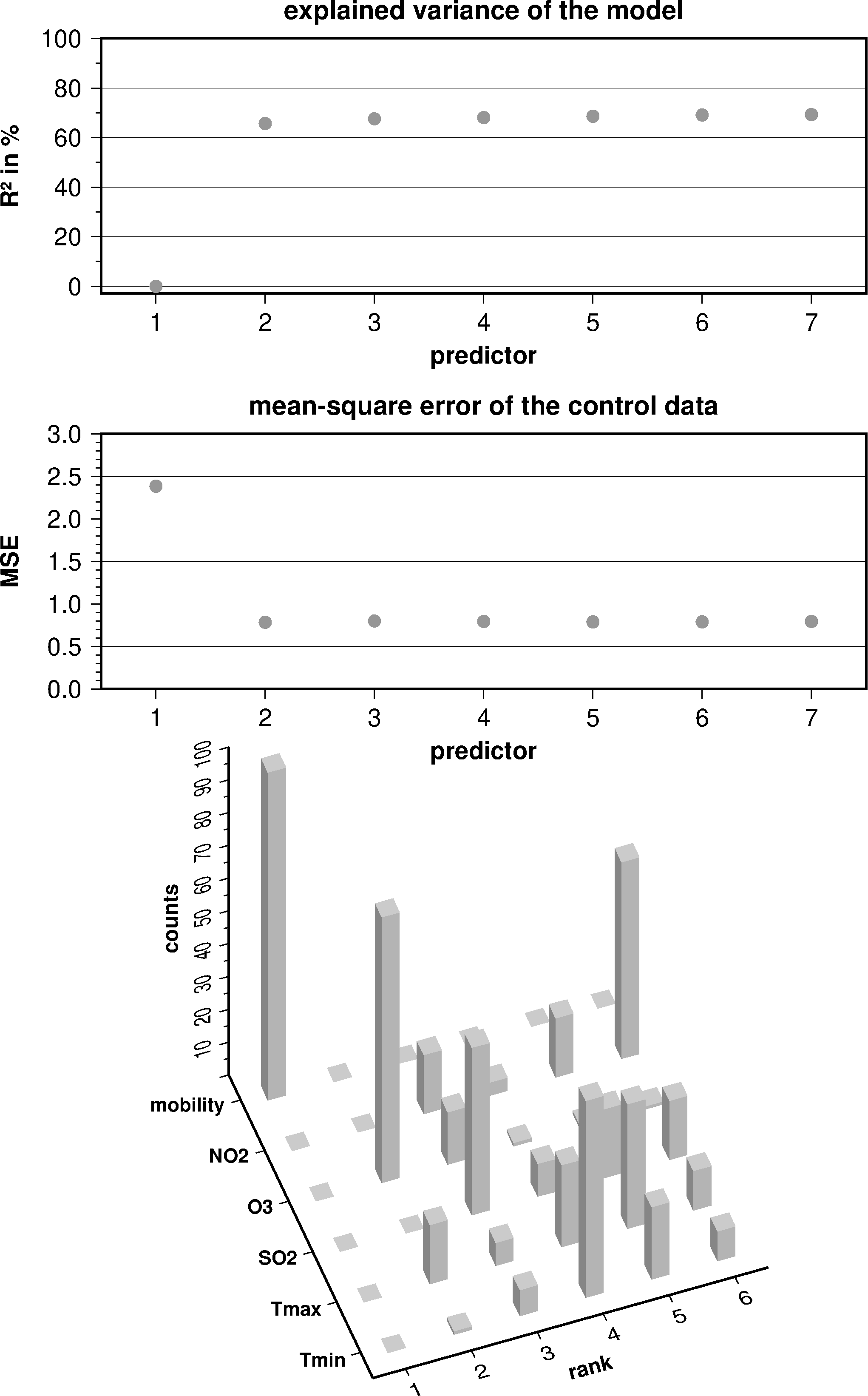


Supplementary Figure S6: In model 6 climate and pollution predictors were squared and all other predictors were kept linearly.


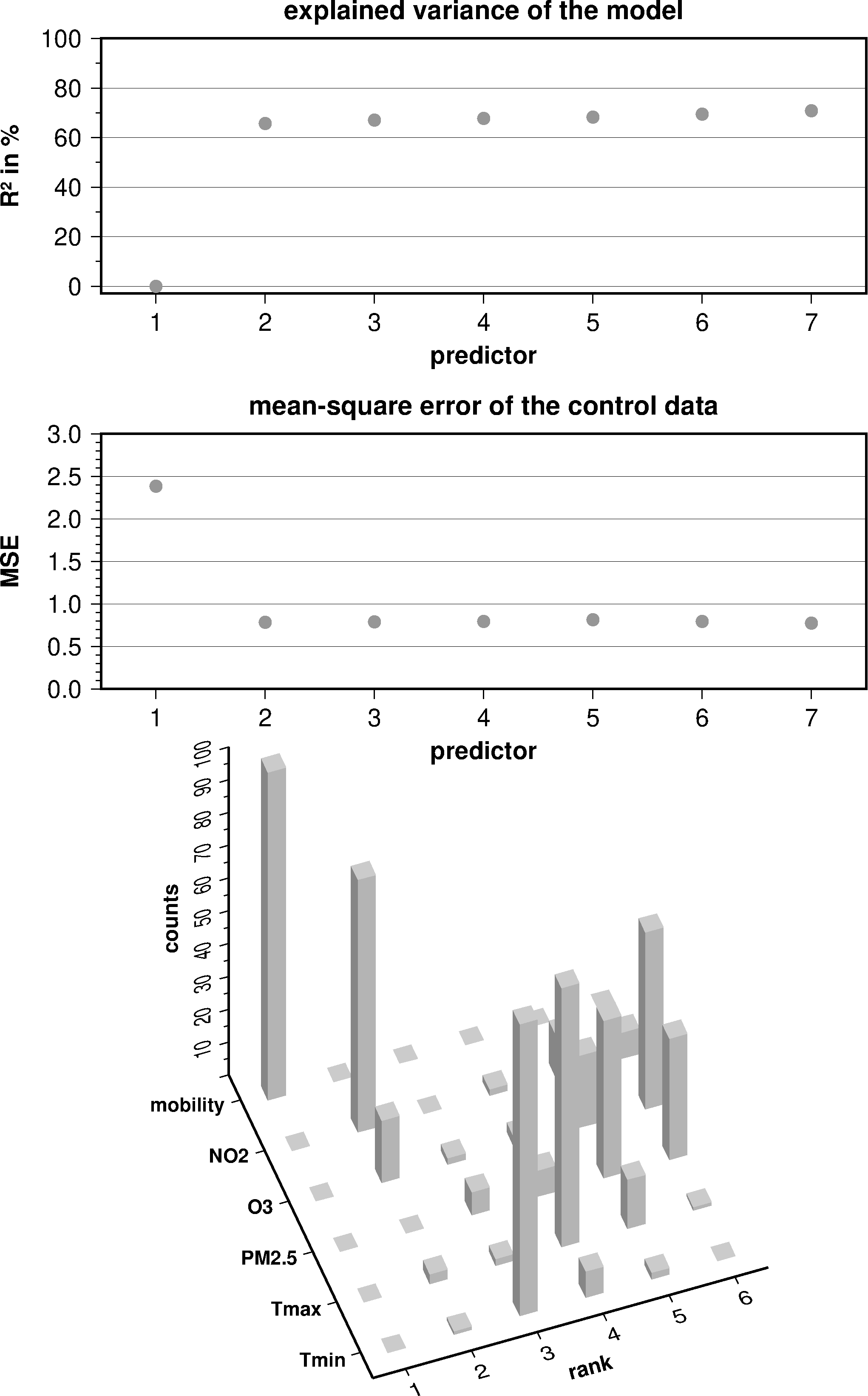


Supplementary Figure S7: In model 7 all predictors were kept linearly, but SO2 was substituted with PM2.5. This model can explain 70.9% of the observed variance but compared to SO2 in model 1 PM2.5 lost two ranks and is now placed on the 5^th^ rank.
